# The value of radiomics to predict abnormal bone mass in type 2 diabetes mellitus patients based on CT imaging for paravertebral muscles

**DOI:** 10.3389/fendo.2022.963246

**Published:** 2022-10-13

**Authors:** Hui Qiu, Hui Yang, Zhe Yang, Qianqian Yao, Shaofeng Duan, Jian Qin, Jianzhong Zhu

**Affiliations:** ^1^ Department of Radiology, The Second Affiliated Hospital of Shandong First Medical University, Tai’an, China; ^2^ GE Healthcare, Precision Health Institution, Shanghai, China

**Keywords:** radiomics, type 2 diabetes mellitus, paravertebral muscles, bone mineral density, computed tomography

## Abstract

**Objective:**

To investigate the value of CT imaging features of paravertebral muscles in predicting abnormal bone mass in patients with type 2 diabetes mellitus.

**Methods:**

The clinical and QCT data of 149 patients with type 2 diabetes mellitus were collected retrospectively. Patients were randomly divided into the training group (n = 90) and the validation group (n = 49). The radiologic model and Nomogram model were established by multivariate Logistic regression analysis. Predictive performance was evaluated using receiver operating characteristic (ROC) curves.

**Results:**

A total of 829 features were extracted from CT images of paravertebral muscles, and 12 optimal predictive features were obtained by the mRMR and Lasso feature selection methods. The radiomics model can better predict bone abnormality in type 2 diabetes mellitus, and the (Area Under Curve) AUC values of the training group and the validation group were 0.94(95% CI, 0.90-0.99) and 0.90(95% CI, 0.82-0.98). The combined Nomogram model, based on radiomics and clinical characteristics (vertebral CT values), showed better predictive efficacy with an AUC values of 0.97(95% CI, 0.94-1.00) in the training group and 0.95(95% CI, 0.90-1.00) in the validation group, compared with the clinical model.

**Conclusion:**

The combination of Nomogram model and radiomics-clinical features of paravertebral muscles has a good predictive value for abnormal bone mass in patients with type 2 diabetes mellitus.

## Introduction

Type 2 diabetes mellitus (T2DM), characterized by hyperglycemia, is a chronic metabolic disease, with many complications. Owing to the problems of aging and high-carbohydrate diet caused by rapid development in China, the incidence of T2DM has been increasing considerably in recent years, with the prevalence rate up to 10.5% (9% ~ 11. 6%) (1) in adults. As a systemic metabolic bone disease, Osteoporosis (OP) can change the bone microstructure and decreased bone mass (2), leading to an increased risk of brittle fractures. In the previous study, T2DM could be associated with bone remodeling and bone turnover impairment, thus resulting in higher risk of OP and brittle fractures for patients with type 2 diabetes. Owing to long-term pain and dysfunction, people’s health would be seriously harmed (3).

The interaction of bones and muscles could be made in the process of metabolism and function. The decrease of bone density and strength is closely related to the metabolic process of muscle denaturation. As an important supporting structure for spines, paravertebral muscle can help to maintain bone density and strength. Some studies (4) have shown that there is a positive correlation between muscle mass and vertebral bone density, and muscle mass of paravertebral muscles is related to the occurrence of vertebral osteoporosis. With the loss of muscle strength, the stability of vertebral body may decrease, which increase the risk of brittle fractures (5). The hyperglycemia and insulin resistance in patients with T2DM could both impair muscle tissue, with the decrease of muscle strength and muscle mass and increase of muscle fat infiltration (6). Compared with normal people, as the disease progressed, the paravertebral muscle mass and vertebral bone mineral density (BMD) decreased in patients with T2DM, and the risk of OP increased significantly. Therefore, to evaluate the relationship between paravertebral muscle group and spinal BMD is important for clinicians to make right decisions.

Quantitative computed tomography (QCT) can accurately measure volume bone mineral density (vBMD) to diagnose OP (7, 8). However, many regional hospitals cannot afford it. In recent years, the studies based on radiomics have been more popular among clinical researchers. Through extracting and analyzing invisible image features from medical images to capture tissue and pathological changes, it is possible to achieve accurate diagnosis and surveillance for diseases (9, 10). The purpose of this study is to evaluate the diagnostic efficacy of paravertebral muscle group characteristics in patients with T2DM to provide a new method for the diagnosis and treatment of osteoporosis.

## Subjects and methods

### Subjects

We retrospectively collected T2DM patients from the Department of Endocrinology, the Second Affiliated Hospital of Shandong First Medical University from June 2021 to December 2022, and record their basic data, including their ages and sexes. The inclusion criteria were:①the 1999 WHO diagnostic criteria for diabetes (11) should be meet for them;②their ages were 35-90. The exclusion criteria were as follows:①patients had other diseases such as endocrine diseases, immune system diseases, chronic inflammatory diseases, malignant tumors;②they had histories of long-term use of calcium, vitamin D and other drugs affecting bone metabolism;③clinical data were not completed. Finally, a total of 149 patients were collected.

### Methods

#### CT examination

All patients underwent T12-L1 vertebral body scans through thoracic or abdominal CT-QCT checks by GE Revolution CT machine. Scanning parameters were: tube voltage 120KV, tube current 355mA, layer thickness and interval 5mm, thin layer 1.25mm, pitch 0.984, tube rotation time 0.8s/rot.

#### Draw the ROI of vertebral body

**Figure 1:**
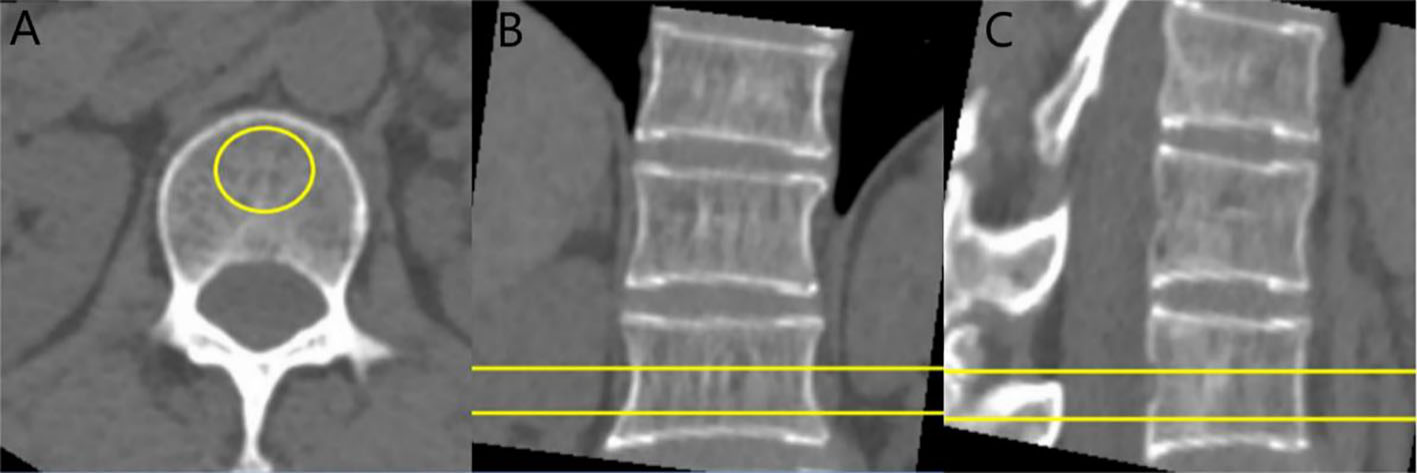
CT value and BMD measurement of vertebral body: cases report. **(A)** axial image, **(B)** sagittal image, and **(C)** coronal image. The ROI is placed on the vertebral cancellous bone, avoiding the vertebral cortical bone and the posterior venous plexus.

The thin slice CT images were transmitted to the post-processing workstation of QCT, and were analyzed by QCT BMD measurement software QCT Pro. Avoiding the cortical bone and posterior venous plexus (**Figure 1**), the region of interest (ROI) was placed in the scope of cancellous bone of T12 and L1 vertebral bodies, with the size of ROI being about 100 mm². and the final value was BMD value of T12 and L1 vertebral bodies. According to the thresholds of 80mg/cm3 and 120mg/cm3 confirmed by American College of Radiology (12), patients were divided into two groups: normal bone mass and abnormal bone mass (including osteopenia and osteoporosis) group.

The CT scan images were transmitted to PACS, and the ROI was delineated in the mediastinum window, and the size of ROI was about 100 mm² (**Figure 1A**). The CT values of the levels of T12, L1 spinous process (avoiding vertebral cortical bone and posterior venous plexus) were measured and the average values were finally got.

##### Delineation and segmentation of paravertebral muscle group ROI

**Figure 2:**
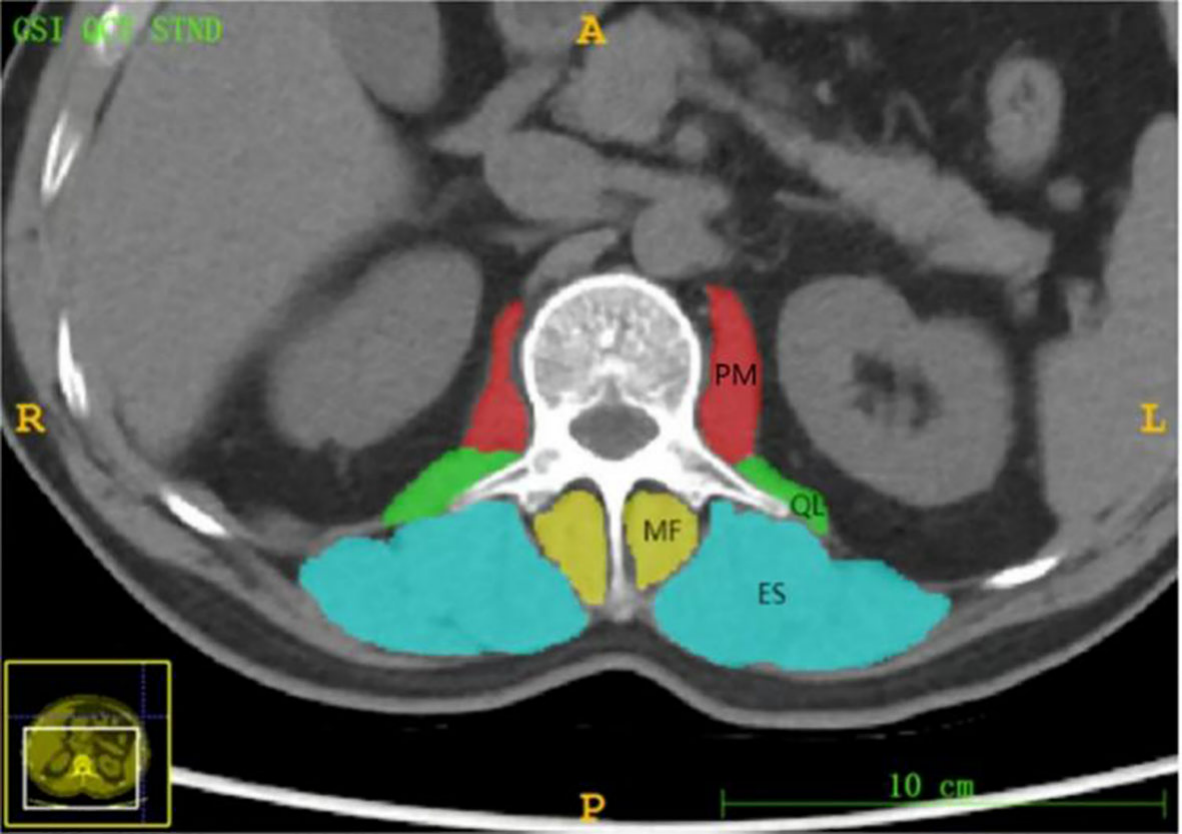
Axial level CT image of the L 1 spinous process. The paravertebral muscles (including the psoas major (PM), quadratus psoas (QL), erector spinalis (ES) and multifidus (MF)) are outlined as shown.

We use the ITK-SNAP software (version 3.6.0, www.itksnap.org) to manually draw the ROI in axial CT images for segmentation. The patient’s CT images were imported in DICOM format to software. Two radiologists with 5 years’ experience in CT diagnosis manually delineated the contours of the spinous process of the L1 vertebral body, the psoas major (PM), psoas quadrates (QL), erector spinalis (ES) and the multifidus (MF) in axial CT images (**Figure 2**) (12). The drawn images with ROI information were saved in NIFTI format for subsequent feature analysis. Images of 40 patients were randomly selected, and two radiologists with more than 5 years of diagnostic qualification performed image segmentation and feature extraction respectively. In order to evaluate the accuracy of ROI segmentation, this study evaluated the consistency by the intra-class correlation coefficient (ICC). When the ICC>0.75, we consider it has a good consistency (13).

#### Radiomic features extraction

Feature extraction was performed using the AK software (AnalysisKit, version 3.2.0, GE Healthcare, China). At first, the images were preprocessed with 3 steps as following: resampling the voxel size into 1×1×1 mm^3^ (14), discretizing the gray values using 25 bin width, and normalizing the gray value before features extraction. Then, 7 kinds of features were extracted, including first-order feature, Shape feature, gray scale dependency matrix feature, gray scale size matrix feature, gray scale run length matrix feature, laplace-transformation (σ= 2, 3) and Wavelet feature.

#### Feature selection and model establishment

The redundancy and correlation of imaging features often lead to over-fitting of prediction models. In this study, minimum-Redundancy Maximum-Relevancy (mRMR) and the least absolute shrinkage and selection operator (LASSO) were used to screen features. First, mRMR was performed to remove redundant and irrelevant features, and then LASSO regression was conducted to select the optimal feature subset to compute the Radscore and construct the final model. For each model, the 10-fold cross validation was used to evaluate the predictive performance of it and to prevent over-fitting.

#### Nomogram model establishment and diagnosis verification

Clinical variables included in this study were age, sex, and T12-L1 vertebral mean CT values. The clinical features were compared by the Pearson chi-square test and analyzed by the multivariate logistic regression. The joint Nomogram model was established based on the imaging and clinical features, and the area AUC under the ROC curve was analyzed by the DeLong Test. The calibration curves of Hosmer-Lemeshow were used to evaluate the nomograms.

#### Statistical analysis

All statistical analyses in this study were performed using R software (version 4.0.2, www.r-project.org), and *p*< 0.05 was considered statistically significant.

## Results

### Clinical characteristics

A total of 149 patients were enrolled in this study and were divided into normal (n = 79) and abnormal (n = 70) bone mass groups according to the criteria published by the American College of Radiology (7). The abnormal bone mass group included patients with osteopenia and osteoporosis. **Table 1** shows the clinical characteristics of the two groups. Patients were randomly divided into training group (n = 90) and verification group (n = 49).

**Table 1:** Basic clinical information about the patients in this study.

		The training group (n=90)			The validation group (n=59)			*p*
		Normal bone mass group	Abnormal bone mass group	*p*	Normal bone mass group	Abnormal bone mass group	*p*	
gender	Male	n = 38	n = 17	0.0004	n = 24	n = 14	0.054	0.8155
	Female	n = 10	n = 25		n = 7	n = 14		
Age		54 ± 9.7	65.5 ± 9.3	<0.05	56.2 ± 8.6	68.1 ± 11.7	<0.05	0.0195
CT value of the vertebrae		154 ± 33.1	104.8 ± 24.3	<0.05	154.4 ± 21.4	102.2 ± 32	<0.05	0.7695

### Selection of imaging features and establishment of Radscore

**Figure 3:**
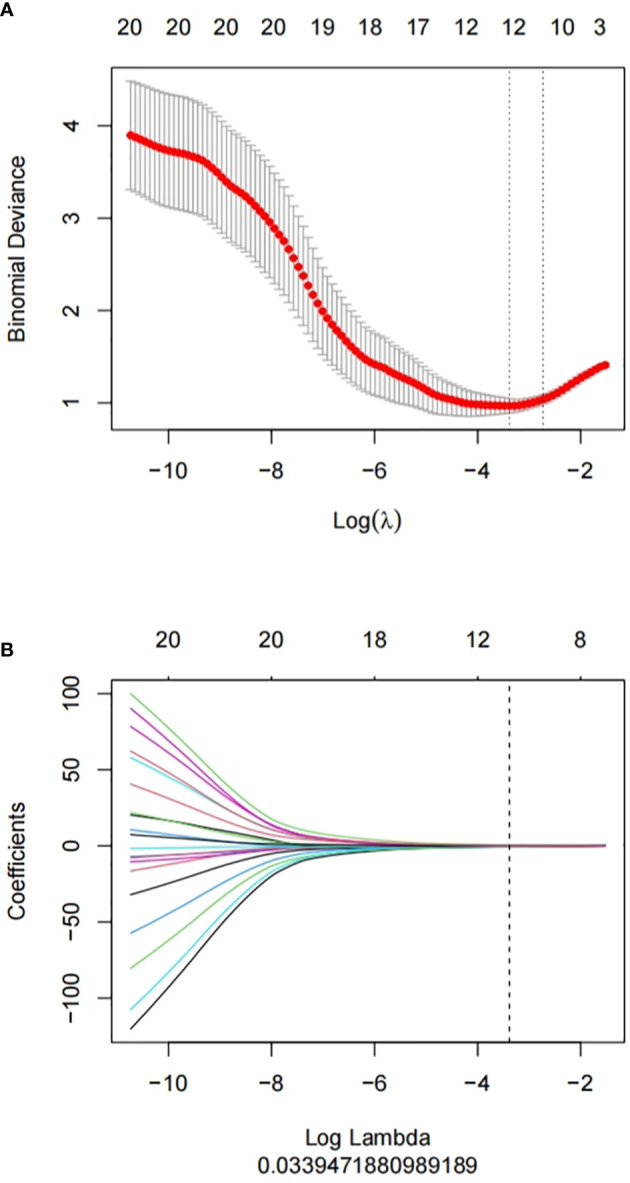
Feature selection proceeded using mRMR and LASSO. **(A)** LASSO method was used to confirm the optimal adjustment parameter λ, and the vertical line was drawn according to the value selected by 10-fold cross-validation. **(B)** the trend lines of model coefficients drawn for 829 imaging features.

**Figure 4:**
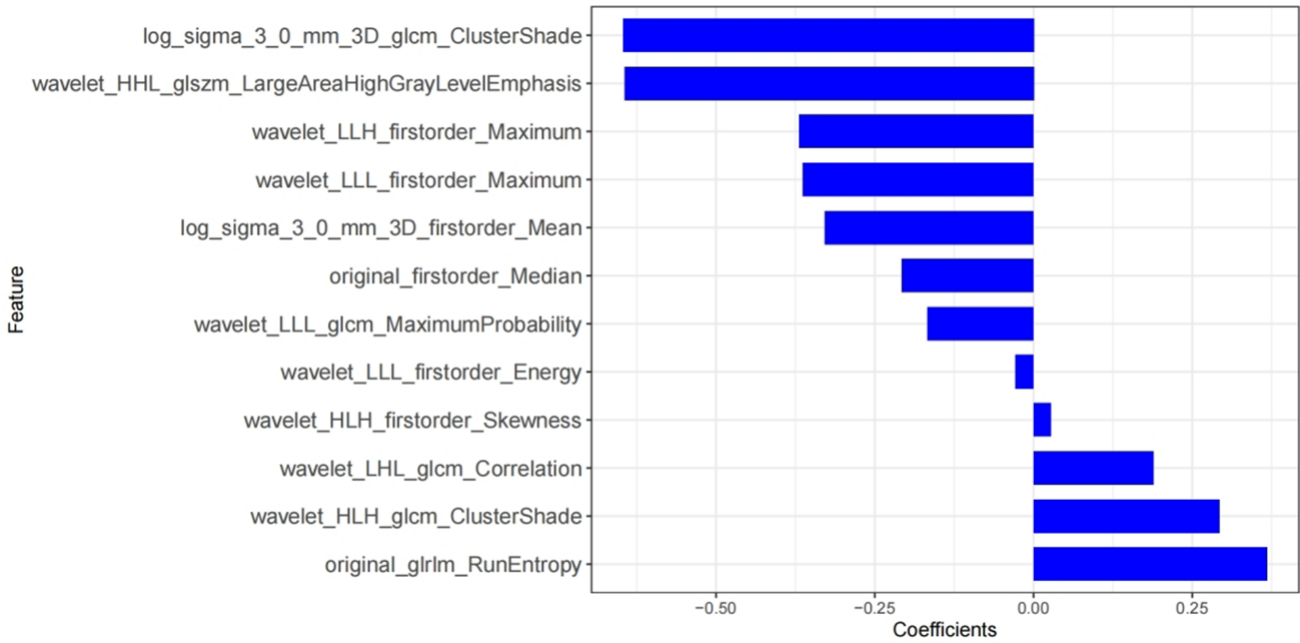
Rad-score histograms, showing 12 texture features with non-zero coefficients on the vertical axis and the radiomics coefficients on the horizontal axis.

AK software was used to extract 829 radiomics features. With ICC, there are 762 features with consistency greater than 0.75. We use mRMR and LASSO for feature selection. Firstly, mRMR was used to remove redundant and irrelevant features, and 30 features were retained. Then the parameter λ was adjusted and tested through the 10-fold cross-validation of LASSO regression. The λ value corresponding to the minimum variance model was selected as the optimal value (λ = 0.034), and the number of features was confirmed (**Figure 3**), a total of 12 subsets of the most predictive features were selected (**Figure 4**) and the corresponding coefficients were calculated; Radscore was calculated by weighting the selected features according to their coefficients. The results were as follows:


$$\begin{array}{l} \text{Radscore}=\;-0.646*\text{log}\_\text{sigma}\_3\_0\_\text{mm}\_3\text{D}\_\text{glcm}\_\text{ClusterShade}\\ \;\;\;\;+0.368*\text{original}\_\text{glrlm}\_\text{RunEntropy}\\ \;\;\;\;+-0.363*\text{wavelet}\_\text{LLL}\_\text{firstorder}\_\text{Maximum}\\ \;\;\;\;+0.027*\text{wavelet}\_\text{HLH}\_\text{firstorder}\_\text{Skewness}\\ \;\;\;\;+-0.207*\text{original}\_\text{firstorder}\_\text{Median}\\ \;\;\;\;+-0.369*\text{wavelet}\_\text{LLH}\_\text{firstorder}\_\text{Maximum}\\ \;\;\;\;+-0.643*\text{wavelet}\_\text{HHL}\_\text{glszm}\_\text{LargeAreaHighGrayLevelEmphasis}\\ \;\;\;\;+-0.329*\text{log}\_\text{sigma}\_3\_0\_\text{mm}\_3\text{D}\_\text{firstorder}\_\text{Mean}\\ \;\;\;\;+-0.028*\text{wavelet}\_\text{LLL}\_\text{firstorder}\_\text{Energy}\\ \;\;\;\;+0.292*\text{wavelet}\_\text{HLH}\_\text{glcm}\_\text{ClusterShade}\\ \;\;\;\;+0.189*\text{wavelet}\_\text{LHL}\_\text{glcm}\_\text{Correlation}\\ \;\;\;\;+-0.167*\text{wavelet}\_\text{LLL}\_\text{glcm}\_\text{MaximumProbability}+\\ \;\;\;\;-0.2 \end{array}$$

**Figure 5:**
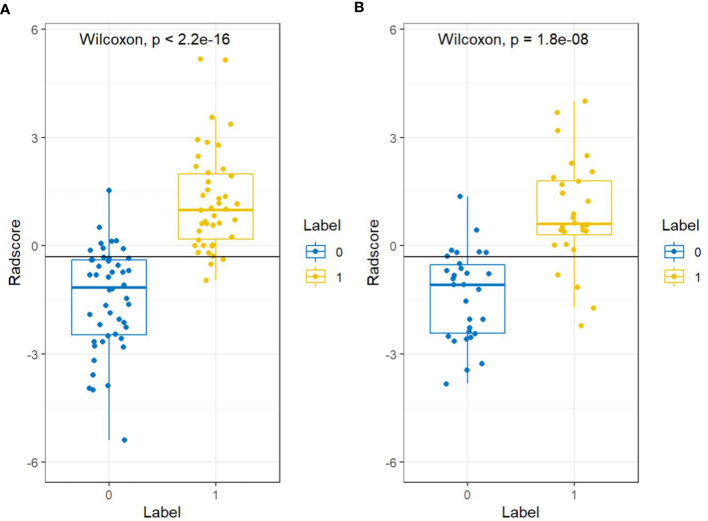
Cross-validation results for the training groups **(A)** and validation groups **(B)**. “0” represents the normal bone mass group and “1” represents the abnormal bone mass group.

**Figure 6:**
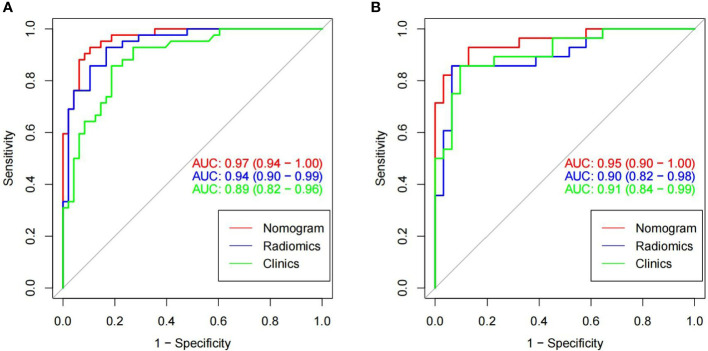
In the training cohorts **(A)** and validation cohorts **(B)**, two images showing ROC curves and AUC values of the combined radiomic-clinical model, the radiomic model and clinical model. In both the training and validation groups, the AUC value of the combined model was higher than the clinical and radiomic models.

There were significant differences in Radscore between training group and validation group (all *p<* 0.001). The results of cross-validation were shown in **Figure 5**. The AUC value of the radiomics model was 0.94(95% CI, 0.90-0.99) in the training group and 0.90(95% CI, 0.82-0.98) in the validation group, and the accuracy, sensitivity, specificity, PPV and NPV were 87.8%, 92.9%, 83.3%, 83.0%, 93.0% and 81.4%, 85.7%, 77.4%, 77.4%, 85.7% in the training group and the verification group (see **Figure 6** and **Table 2**).

**Table 2:** In the training group and the validation group, a table showing the diagnostic efficacy of radiomic, clinical, and radiomic-clinical joint models.

Model		Accuracy	Sensitivity	Specificity	Pos.Pred.Value	Neg.Pred.Value	AUC (95%CI)	P-value of DeLong-Test(vsClinics)
Radiomics	Training	0.878	0.929	0.833	0.830	0.930	0.94 (0.90-0.99)	0.1607
	Validation	0.814	0.857	0.774	0.774	0.857	0.90 (0.82-0.98)	0.8136
Clinics	Training	0.833	0.857	0.813	0.800	0.867	0.89 (0.82-0.96)	
	Validation	0.831	0.750	0.903	0.875	0.800	0.91 (0.84-0.99)	
Nomogram	Training	0.911	0.929	0.896	0.886	0.935	0.97 (0.94-1.00)	0.0065
	Validation	0.881	0.889	0.875	0.857	0.903	0.95 (0.90-1.00)	0.1775

### Construction of nomogram and its diagnostic validation

**Figure 7:**
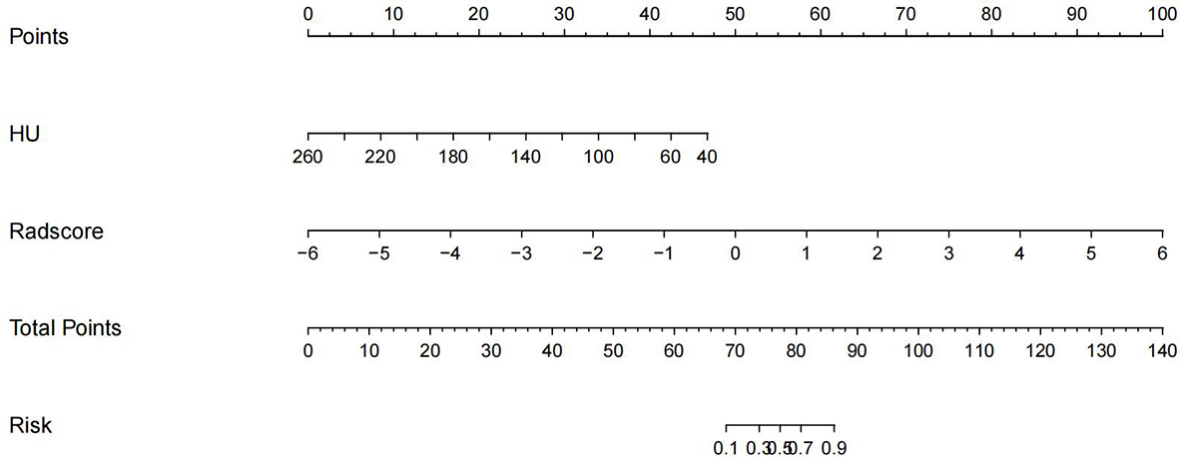
A nomogram of imaging-clinical joint model, including CT values of the vertebral body and Radscore.

**Figure 8:**
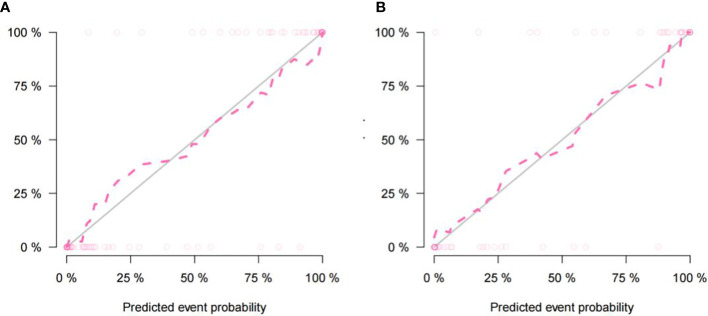
The nomogram calibration curve of the training cohort **(A)** and the validation cohort **(B)** showing the relationship between the predictive and true values. The distance between the dotted line and the solid line indicated the prediction ability of the model, with an inverse relationship between the distance and prediction ability.

The imaging and clinical features were compared by the Pearson chi-square test. When the differences were statistically significant, a combined radiomic-clinical Nomogram model was established by the multivariate logistic regression analysis (**Figure 7**), with the nomo-score = (Intercept)* 6.92713186590545 + HU* -0.0527666121405694 + Radscore* 2.07053832723835. The Hosmer-Lemeshow curve was used to calibrate Nomogram and showed no significant difference between the training and validation groups (*p* = 0.967 and 0.475)(**Figure 8**). The AUC values of this Nomogram model in training group is 0.97(95%CI, 0.94-1.00), with accuracy sensitivity and specificity of 91.1%, 92.9% and 89.6%, respectively. In the validation group, the Nomogram model also shows a good prediction performance with an AUC value of 0.95(95%CI, 0.90-1.00), accuracy sensitivity and specificity of 88.1%, 88.9%, 87.5% (**Figure 6** and **Table 2**). There were no significant differences between the radiomic model and the clinical model (*p* = 0.1607 and 0.8136). Compared with the clinical model AUC, the combined Nomogram model showed significant difference in the training group (*p* = 0.0065,< 0.05), without a significant difference in the validation group (*p* = 0.1775) (see **Table 2**).

## Discussion

In this study, the Nomogram model, established based on radscore and vertebral CT values, has higher diagnostic efficacy than the radiography model and clinical model. For the T2DM patients, after CT scans, this model can accurately differentiate BMD. Therefore, it can be considered as a good method to diagnose abnormal bone mass for patients with type 2 diabetes mellitus, especially for those patients with normal BMD values who originally need further examinations.

The changes of paravertebral muscles, causes by hyperglycaemia and insulin resistance in T2DM patients, can affect glucose metabolism. As an important target organ of insulin action, skeletal muscles, being crucial to keep the glucose content steadily, can be involved in the more than 80% process of this kind of the metabolism. The insulin resistance in these muscles can lead to the decrease of muscle mass and strength (14). Some studies have shown that ectopic fat deposition may be the reason for insulin resistance. Owing to some mechanisms, like the changes of bone microstructures by hyperglycemia, chronic inflammation and accumulation of advanced glycation end products, the osteoblast metabolism and maturation can be harmed (15), resulting in lower muscle mass, strength and bone mineral density in these patients. Other studies have shown that osteoporosis and paraspinal muscle degeneration may interact and coexist in patients with degenerative lumbar disorders (16).

In recent years, the relationship between paravertebral muscles and vertebral BMD has become a hotspot for studies, and mechanical and metabolic relationships between skeletal muscle and bone have also been proposed. Muscles and bones interact at the organ, cellular and molecular levels through bi-directional pathways. Mechanical load and muscle contraction are the most important factors for bone mass and shape (12, 17). Exerting mechanical load on bone through muscle contraction, skeletal muscles can promote bone metabolism and inhibit the reduction of bone mass and mineral density. Therefore, the relationship between paravertebral muscles and vertebral BMD should be focused on in the treatment.

At present, to measure the CT value of vertebral cancellous is the most commonly method to screen for opportunistic osteoporosis. Therefore, it is reasonable to study combining the Radiomics characteristics of paravertebral muscle group with vertebral HU value. Zouda et al. (18) used vertebral CT values to screen for osteoporosis in patients with lumbar Degenerative disease, showing high specificity; Cohen et al. (19) conducted a validation study of routine CT screening for opportunistic osteoporosis in a multi-ethnic population in the Middle East, showing a significant correlation between vertebral CT values and BMD, They suggested that when CT values were< 110 HU, BMD was needed to be examined for patients. In this study, the validity of the vertebral CT value model was similar to previous studies. In addition, the Deron Test and ROC curve showed that the radiologic model performed better than the clinical model.

Radiomics analysis based on conventional chest and/or abdominal CT scans can provide an alternative for osteopathia screening in patients with T2DM. As a quantitative high-precision image analysis technique, Radiomics has the potential to discover invisible disease features, enabling accurate disease diagnosis and monitoring (20). However, the process of radiomics analysis was currently complicated. As studies on automatic segmentation progress, it is possible to integrate feature extraction and screening into a single software program. It can be achieved by one button. This study suggests that radiomic analysis based on CT scans was an effective screening method for bone abnormalities.

However, our study has several limitations. Firstly, this study was a single-center study with a small sample size, and patients were not grouped by the BMD values. Secondly, in this study, the DeLong test was made between ROC curves of the joint model and the clinical model, showing *p* > 0.05 in the validation group, but it was not a reliable result owing to the small sample size. In addition, more clinical features could be selected in future studies. Finally, only three levels selected to draw ROI may not represent the whole muscle. To improve the accuracy of the model, the ROI range should be increased.

In summary, with the data of vertebral CT values and radiomics features for paraspinal muscle group, the joint model has a good performance in predicting the changes of bone mass for T2DM patients, providing a good method for clinical decision.

## Data availability statement

The original contributions presented in the study are included in the article/Supplementary Material. Further inquiries can be directed to the corresponding authors.

## Ethics statement

The studies involving human participants were reviewed and approved by Ethics Committee/IRB of The Second Affiliated Hospital of Shandong First Medical University. The patients/participants provided their written informed consent to participate in this study.

## Author contributions

HQ, HY, and JQ designed the study. HQ and SD performed the data analysis. HY, JQ, and QQY contributed to the data interpretation. HQ wrote the manuscript. HY, ZY, and JQ made contributions to its final form. All authors have read and approved the manuscript in this form.

## Funding

This study was funded by Academic Promotion Programme of Shandong First Medical University (No. 2019QL017) and Tai’an City Science and Technology Development Plan (No. 2021NS254).

## Acknowledgments

Thanks to Peizhe Wang for his earnest efforts in establishing the final research method. In addition, We apologize to those authors we have not been able to cite due to space constraints.

## Conflict of interest

Author SD was employed by GE Healthcare.

The remaining authors declare that the research was conducted in the absence of any commercial or financial relationships that could be construed as a potential conflict of interest.

## Publisher’s note

All claims expressed in this article are solely those of the authors and do not necessarily represent those of their affiliated organizations, or those of the publisher, the editors and the reviewers. Any product that may be evaluated in this article, or claim that may be made by its manufacturer, is not guaranteed or endorsed by the publisher.
